# Method for appraising model validity of randomised controlled trials of homeopathic treatment: multi-rater concordance study

**DOI:** 10.1186/1471-2288-12-49

**Published:** 2012-04-17

**Authors:** Robert T Mathie, Helmut Roniger, Michel Van Wassenhoven, Joyce Frye, Jennifer Jacobs, Menachem Oberbaum, Marie-France Bordet, Chaturbhuja Nayak, Gilles Chaufferin, John A Ives, Flávio Dantas, Peter Fisher

**Affiliations:** 1 British Homeopathic Association, Hahnemann House, 29 Park Street West, Luton LU1 3BE, UK; 2 Royal London Hospital for Integrated Medicine, 60 Great Ormond Street, London WC1N 3HR, UK; 3 LMHI Research Secretariat, Rue Taille Madame 23, B-1450 Chastre, Belgium; 4 Center for Integrative Medicine, University of Maryland School of Medicine, Baltimore, MD 21201, USA; 5 School of Public Health and Community Medicine, University of Washington, Seattle, WA 98195, USA; 6 Center for Integrative Complementary Medicine, Shaare Zedek Medical Center, Jerusalem, Israel; 7 Boiron, 20 Rue de la Liberation, 69110 Sainte Foy-lès-Lyon, France; 8 (Formerly) Central Council for Research in Homeopathy, Department of AYUSH, Ministry of Health & Family Welfare, Government of India, New Delhi 110058, India; 9 Samueli Institute, 1737 King Street (Suite 600), Alexandria, VA 22314, USA; 10 Department of Clinical Medicine, Universidade Federal de Uberlândia, Uberlândia, Brazil

## Abstract

**Background:**

A method for assessing the model validity of randomised controlled trials of homeopathy is needed. To date, only conventional standards for assessing intrinsic bias (internal validity) of trials have been invoked, with little recognition of the special characteristics of homeopathy. We aimed to identify relevant judgmental domains to use in assessing the model validity of homeopathic treatment (MVHT). We define MVHT as the extent to which a homeopathic intervention and the main measure of its outcome, as implemented in a randomised controlled trial (RCT), reflect 'state-of-the-art' homeopathic practice.

**Methods:**

Using an iterative process, an international group of experts developed a set of six judgmental domains, with associated descriptive criteria. The domains address: (I) the rationale for the choice of the particular homeopathic intervention; (II) the homeopathic principles reflected in the intervention; (III) the extent of homeopathic practitioner input; (IV) the nature of the main outcome measure; (V) the capability of the main outcome measure to detect change; (VI) the length of follow-up to the endpoint of the study. Six papers reporting RCTs of homeopathy of varying design were randomly selected from the literature. A standard form was used to record each assessor's independent response per domain, using the optional verdicts 'Yes', 'Unclear', 'No'. Concordance among the eight verdicts per domain, across all six papers, was evaluated using the kappa (κ) statistic.

**Results:**

The six judgmental domains enabled MVHT to be assessed with 'fair' to 'almost perfect' concordance in each case. For the six RCTs examined, the method allowed MVHT to be classified overall as 'acceptable' in three, 'unclear' in two, and 'inadequate' in one.

**Conclusion:**

Future systematic reviews of RCTs in homeopathy should adopt the MVHT method as part of a complete appraisal of trial validity.

## Background

In systematic reviews, the criteria for defining the quality of a randomised controlled trial (RCT), as defined by *QUOROM *[1] and *PRISMA *[2] and adopted by the Cochrane Collaboration [3], are designed to appraise internal validity (the extent to which the design, conduct and analysis of a trial has minimised or avoided biases in its comparison of treatments [4-6]). The external validity of an RCT (the extent to which the study's results provide a correct basis for generalisation to other clinical circumstances [4-6]) is also important but is less often addressed by such formal assessment. It is difficult to optimise both attributes in a single RCT: an explanatory trial focuses mainly on internal validity, whereas a pragmatic trial is designed to ensure maximum external validity. Nevertheless, if the results of an RCT are not internally valid, the question of external validity may be regarded as irrelevant; equally, the results of an internally valid RCT may be clinically limited if it lacks external validity [5].

In the field of complementary/alternative medicine (CAM), it is recognised that a third attribute of an RCT - its model validity - is also of particular importance [7,8]. Model validity reflects the concordance between the trial study design and "state of the art" practice for the intervention under investigation [4]. In their discussion of RCT methods in CAM, Verhoef and co-authors go farther, advising that model validity should "encompass ... the unique healing theory and therapeutic context of the intervention" [7].

In reviewing RCTs in homeopathy, internal validity has been appraised using either Jadad scores [9] or Cochrane-based criteria [10]. The external validity of this body of literature has also been addressed [11]. However, the 'homeopathicity' [12] of the therapeutic modality and of the clinical outcome measure/s used (i.e. the key characteristics of model validity) have never been formally accounted for in the assessment of trial quality. Some homeopathy trials have been broadly criticised for their lack of acceptable model validity, e.g.: homeopathic complex medication in female infertility [13,14]; individualised homeopathic treatment of childhood asthma [15,16]; isopathic treatment of hay fever [17,18]. Recognising that the characteristics of the homeopathic intervention and outcome measures are important seems a prerequisite for developing the most refined and relevant approaches to systematic review in this field of clinical research.

A set of guidelines (*RedHot*) [19] has established criteria for reporting data on homeopathic treatments; a similar set of reporting criteria is available for clinical trials of acupuncture (*STRICTA*) [20]. Recommendations of this nature are important since they require reporting of methodological details not addressed by generic standards such as *CONSORT *[21]. Nevertheless, the *RedHot *guidelines for homeopathy do not provide the means to define or assess model validity.

The work of Verhoef and colleagues highlights the importance of model validity, and they offer four broad headings under which assessment of homeopathy RCTs might take place: "representativeness"; "equipoise and credibility"; "model congruity"; "context" [7]. These attributes are a suitable reflection of the key characteristics of homeopathy that must be assessed for model validity, but they do not provide a direct means to undertake a practical formal assessment. The importance of such undertaking has been emphasised in two papers by Bornhöft and colleagues [4,22], who expressed concern that the current research literature - and thus the systematic review of it - may contain high risk of false negative results.

We addressed this issue by creating and testing a practical set of judgmental domains and accompanying criteria that may be used, within systematic review, to appraise RCTs for model validity of homeopathic treatment (MVHT). For the purpose of this work, we define MVHT as the extent to which a homeopathic intervention and the main measure of its outcome, as implemented in an RCT, reflect 'state-of-the-art' clinical practice in homeopathy [4].

## Methods and results

### Preliminary set of domains

A set of six domains for assessing MVHT was drafted, based on the checklist proposed by Bornhöft et al. [4]. This draft was considered, round a conference table, by the full membership of the International Scientific Committee for Homeopathic Investigations (ISCHI) during its meeting in October 2010, Paris, France. Each member of the committee is an experienced homeopathic practitioner and/or researcher. Following detailed discussion amongst the group, a set of draft assessment domains was proposed as follows:

***Domain I: ***Is the condition amenable to homeopathic intervention?

***Domain II: ***Is the specific intervention used consistent with homeopathic principles?

***Domain III: ***Would the rationale for the intervention used be supported by a significant body of homeopathic practitioners?

***Domain IV: ***Does the main outcome measure reflect the key effects expected of the intervention used?

***Domain V: ***Is the main outcome measure capable of detecting change?

***Domain VI: ***Is the length of follow-up for the main outcome appropriate to detect the intended effect of the intervention used?

Based on these six draft domains, the 10 group members who are homeopathic practitioners/researchers (representing Europe [n = 5], USA, [n = 3], Brazil [n = 1] and India [n = 1]) then participated in appraising relevant methodological facts about three placebo-controlled RCTs of homeopathic treatment published in the peer-reviewed literature [23-25]. The following summary facts were provided per trial: nature of the homeopathic intervention (treatment modality; potency of medicine); main outcome measure and its timing. Full texts of the papers were not supplied. The trials were selected to represent at least one example of individualised (classical) homeopathy and at least one example of non-individualised homeopathy (the same single/complex homeopathic medicine for every trial participant).

Each ISCHI member independently assessed each trial, recording their verdicts on each of the above six domains with the answers 'Yes', 'No', or 'Unclear'. Those judgments were then shared with the entire group and the information (anonymised) noted on a flip-chart. Members did not vote on any RCT in which he/she is a co-author.

The majority views were:

Ferley [23]. RCT of *Oscillococcinum *in influenza was clearly acceptable in five of the six domains, but was unclear as regards the homeopathic nature of the intervention (domain II).

Jacobs [24]. RCT of individualised homeopathy for childhood diarrhoea was acceptable in all domains of MVHT.

Schmidt [25]. RCT of *Thyroidinum *for body weight reduction was inadequate in domain I and domain VI and was unclear in domains II-V.

### Refined set of domains and judgmental criteria

Some important discrepancies of interpretation between assessors were acknowledged at the above meeting and so the domains and their associated judgmental criteria were refined subsequently. The aim was to determine a set of domains and criteria whose style mirrors that used by the Cochrane Collaboration for assessing risk-of-bias (internal validity) [3].

From further discussion within the ISCHI membership and with other colleagues (see *Acknowledgements*), six revised domains were agreed upon. These comprised the following wording (with associated judgmental criteria provided per domain - details in Table 1):

***Domain I (Rationale): ***Would a significant body of accredited homeopaths support the rationale for the intervention used in the study?

***Domain II (Principles): ***Is the specific intervention used consistent with homeopathic principles?

***Domain III (Practitioner): ***Does the study have suitably qualified and experienced homeopathic practitioner input?

***Domain IV (Outcome measure): ***Does the main outcome measure reflect the main effect expected of the intervention used?

***Domain V (Sensitivity): ***Is the main outcome measure capable of detecting change?

***Domain VI (Follow-up): ***Is the length of follow-up for the main outcome measure appropriate to detect the intended effect of the intervention?

**Table 1:** Refined domains and criteria used to assess RCTs for model validity of homeopathic treatment (MVHT)^a^

**Domain I *(Rationale)***. **Would a significant body of accredited homeopaths support the rationale for the intervention used in the study? **^b, c^	
**Criteria for a judgment of 'Yes' (i.e. acceptable MVHT for this domain)**	*Both of the following:* • Clinical knowledge and practice inform that, for the condition under investigation, the health of patients may be benefited by homeopathic intervention. • A substantial number of experienced homeopaths would support the choice of this intervention for this type of patient.

**Criteria for a judgment of 'No' (i.e. inadequate MVHT for this domain)**	*One or both of the following:* • Clinical knowledge and practice do not inform that, for the condition under investigation, the health of patients may be benefited by homeopathic intervention. • A substantial number of experienced homeopaths would not support the choice of this intervention for this type of patient.

**Criteria for a judgment of 'Unclear' (i.e. uncertain MVHT for this domain)**	*One of the following:* • There are likely to be important differences amongst experienced homeopathic opinion on the rationale for the intervention used. • Insufficient information to permit judgment of 'Yes' or 'No'.

**Domain II *(Principles)*. Is the specific intervention used consistent with homeopathic principles?**	

**Criteria for a judgment of 'Yes' (i.e. acceptable MVHT for this domain)**	*One or both of the following:* • The intervention used is based on the principle of 'like treats like' or it is based on the principle of isopathy (i.e. use of homeopathic biotherapy, endogenous molecule or aetiological substance). • Literature sources (materia medica, repertory, etc.) are available that convincingly justify the specific intervention.

**Criteria for a judgment of 'No' (i.e. inadequate MVHT for this domain)**	*One or both of the following:* • The intervention used is not based on the principle of 'like treats like' or it is not based on the principle of isopathy (i.e. use of homeopathic biotherapy, endogenous molecule or aetiological substance). • Literature sources do not convincingly justify the specific intervention.

**Criterion for a judgment of 'Unclear' (i.e. uncertain MVHT for this domain)**	• Insufficient information to permit judgment of 'Yes' or 'No'.

**Domain III *(Practitioner)*. Does the study have suitably qualified and experienced homeopathic practitioner input?**	

**Criteria for a judgment of 'Yes' (i.e. acceptable MVHT for this domain)**	*Either of the following, as appropriate: *^d^ • Individualised homeopathy: Those who have prescribed the homeopathic medicine(s) in this case are suitably trained^e ^and experienced in homeopathy to manage the condition under investigation. • Non-individualised homeopathy: There is evidence that experienced homeopathic input (and/or a suitable literature source - e.g. materia medica, repertory) has been involved in informing the choice of the medicine(s) used commonly for all patients in the study.

**Criteria for a judgment of 'No' (i.e. inadequate MVHT for this domain)**	*Either of the following, as appropriate:* • Individualised homeopathy: Those who have prescribed the homeopathic medicine(s) in this case are not suitably trained and experienced in homeopathy to manage the condition under investigation. • Non-individualised homeopathy: There is evidence that experienced homeopathic input (or a suitable literature source - e.g. materia medica, repertory) has not been involved in informing the choice of the medicine(s) used commonly for all patients in the study.

**Criterion for a judgment of 'Unclear' (i.e. uncertain MVHT for this domain)**	• Insufficient information to permit judgment of 'Yes' or 'No'.

**Domain IV *(Outcome measure)*. Does the main outcome measure reflect the main effect expected of the intervention used?**	

**Criterion for a judgment of 'Yes' (i.e. acceptable MVHT for this domain)**	• The main clinical effect expected of the intervention is adequately measured by the main outcome used.

**Criterion for a judgment of 'No' (i.e. inadequate MVHT for this domain)**	• The main clinical effect expected of the intervention is not adequately measured by the main outcome used.

**Criterion for a judgment of 'Unclear' (i.e. uncertain MVHT for this domain)**	• Insufficient information to permit judgment of 'Yes' or 'No'.

**Domain V *(Sensitivity)*. Is the main outcome measure capable of detecting change?**	

**Criteria for a judgment of 'Yes' (i.e. acceptable MVHT for this domain)**	*All of the following:* • The main outcome measure is sensitive to changes of the magnitude expected in the patients under investigation. • The main outcome measure is capable of determining both improvement and deterioration. • The main outcome measure shows no evidence of a 'floor effect' and/or 'ceiling effect'.^f^

**Criteria for a judgment of 'No' (i.e. inadequate MVHT for this domain)**	*Any one or more of the following:* • The main outcome measure is not sensitive to changes of the magnitude expected in the patients under investigation. • The main outcome measure is not capable of determining both improvement and deterioration. • The main outcome measure shows evidence of a 'floor effect' and/or 'ceiling effect'.

**Criterion for a judgment of 'Unclear' (i.e. uncertain MVHT for this domain)**	• Insufficient information to permit judgment of 'Yes' or 'No'.

**Domain VI *(Follow-up)*. Is the length of follow-up for the main outcome measure appropriate to detect the intended effect of the intervention?**	

**Criterion for a judgment of 'Yes' (i.e. acceptable MVHT for this domain)**	• The time-point selected for main follow-up measurement provides sufficient opportunity for a clinical change to be observed.

**Criterion for a judgment of 'No' (i.e. inadequate MVHT for this domain)**	• The time-point selected for main follow-up measurement does not provide sufficient opportunity for a clinical change to be observed.

**Criterion for a judgment of 'Unclear' (i.e. uncertain MVHT for this domain)**	• Insufficient information to permit judgment of 'Yes' or 'No'.

^a^Given the prior assessment that medicines used were prepared according to homeopathic pharmaceutical practice

^b^The assessment should reflect whether the rationale for the intervention could feasibly apply to *each and every *patient studied

^c^A novel homeopathic medicine might be eligible to fulfil the criteria for 'Yes' in circumstances where the toxicology or proving symptoms support its use, according to the Principle of Similars, in the study sample

^d^The initially refined Domain III did not include the criteria sub-divisions 'Individualised homeopathy' and 'Non-individualised homeopathy'. Some difficulty resulted initially in the ensuing assessments, and so the papers were reappraised, for Domain III only, with the subdivisions added (with the consequent findings as reported in Table 3 and Table 4)

^e^'Suitably trained': To enable this assessment, the experience, accreditation and qualifications of the practitioners must be stated in the original paper (as per the *RedHot *guidelines - *Homeopathy *2007; **96**:42-45)

^f^The outcome measure does not reach a minimum (i.e. 'floor effect') and/or a maximum (i.e. 'ceiling effect') value, making it incapable of detecting change across the entire clinically meaningful range of the study sample

To characterise and test this refined method in practice, another six RCTs of homeopathic treatment were selected randomly from the peer-reviewed journal literature, ensuring that individualised homeopathy was the subject of investigation in at least two: five were placebo-controlled [26-30], and one used active control [31]. Eight members of our group then appraised these RCTs, based on full texts of the six papers; summary information about each trial was also provided to them (Table 2) by the study coordinator (RTM). The results reported in each paper were not taken into account in the assessment process, though the assessors were not formally blinded to the Results or Discussion sections in each case. In papers where a single primary outcome had not been pre-defined by a trial's authors, a 'main' outcome measure was identified for that trial using the hierarchical structure given in the WHO International Classification of Functioning, Disability and Health [32]: for the six papers examined, a single primary outcome had not been pre-defined in three cases [26-28].

**Table 2:** Summary information per RCT assessed for MVHT using refined set of domains and judgmental criteria

Paper	Attributes of RCT				
**First author, year**	**Medical condition studied**	**Homeopathic intervention**	**Comparison group**	**'Main' outcome measure**	**Length of follow-up**

Lewith, 2002 [26]	Allergic asthma	House dust mite	Placebo	Asthma VAS	16 wk

Kainz, 1996 [27]	Warts	Individualised (from 10 options)	Placebo	Responders: patients with at least 50% reduction in area of skin affected by warts	8 wk

McCutcheon, 1996 [28]	Anxiety	Combination (9 medicines)	Placebo	State Anxiety score	15 d

Bell, 2004 [29]	Fibromyalgia	Individualised	Placebo	Tender point pain on palpation	3 mo

Robertson, 2007 [30]	Post-operative pain	Arnica montana	Placebo	Change in tonsillectomy pain score (VAS)	14 d

Adler, 2009 [31]	Depression	Individualised	Fluoxetine	Change in MADRS score from baseline	8 wk

'Main' outcome measure was identified per RCT using the WHO International Classification of Functioning, Disability and Health, 2002 [32]

*MADRS *Montgomery & Åsberg Depression Rating Scale

*VAS *Visual Analogue Scale

The eight assessors submitted their independent verdicts, via e-mail on a standard form, to the study coordinator. The total numbers of verdicts ('Yes', 'Unclear', 'No') recorded per domain are listed in Table 3. The table also shows the concordance among the eight assessors in their verdict per domain. This is presented using Fleiss's normalised measure of overall multi-rater agreement, corrected for the amount expected by chance: the kappa (κ) statistic [33], calculated using Microsoft *Excel*. The value of κ (whose arithmetic range is 0 [no agreement] to 1 [perfect agreement]) varied from 0.42 (domain III) to 0.94 (domain V): see Table 3.

**Table 3:** Verdicts for domains I-VI, indicating number of assessors rating 'Yes', 'Unclear', or 'No' per paper, and the kappa statistic (κ) per domain

Domain I *(Rationale)* κ = 0.50			
**Paper**	**Yes**	**Unclear**	**No**

Lewith	3	1	4

Kainz	6	2	0

McCutcheon	2	4	2

Bell	8	0	0

Robertson	6	1	1

Adler	8	0	0

**Domain II *(Principles)*** **κ = 0.68**			

**Paper**	**Yes**	**Unclear**	**No**

Lewith	7	1	0

Kainz	7	1	0

McCutcheon	2	5	1

Bell	8	0	0

Robertson	6	0	2

Adler	8	0	0

**Domain III *(Practitioner)*** **κ = 0.42**			

**Paper**	**Yes**	**Unclear**	**No**

Lewith	6	1	1

Kainz	5	3	0

McCutcheon	1	4	3

Bell	8	0	0

Robertson	4	2	2

Adler	7	1	0

**Domain IV *(Outcome measure)*** **κ = 0.92**			

**Paper**	**Yes**	**Unclear**	**No**

Lewith	6	2	0

Kainz	8	0	0

McCutcheon	7	1	0

Bell	8	0	0

Robertson	7	0	1

Adler	8	0	0

**Domain V *(Sensitivity)*** **κ = 0.94**			

**Paper**	**Yes**	**Unclear**	**No**

Lewith	8	0	0

Kainz	8	0	0

McCutcheon	4	3	1

Bell	8	0	0

Robertson	7	1	0

Adler	8	0	0

**Domain VI *(Follow-up)*** **κ = 0.43**			

**Paper**	**Yes**	**Unclear**	**No**

Lewith	5	1	2

Kainz	4	4	0

McCutcheon	3	3	2

Bell	8	0	0

Robertson	8	0	0

Adler	6	1	1

Table 4 presents the same verdicts as Table 3, but focused per paper to highlight the majority verdict per domain. The most common majority verdict was 'Yes', which occurred in a total of 30 out of 36 instances. One trial (Lewith [26]) attained a majority of 'No' in one domain; two trials (Kainz [27], McCutcheon [28]) each contained one or more 'Unclear' in the majority verdicts; the remaining three trials (Adler [31], Bell [29], Robertson [30]) each comprised a full complement of six 'Yes' majority verdicts. Table 4 also itemises the overall majority verdict per paper (reflecting number of domains with majority responses 'Yes', 'Unclear', 'No').

**Table 4:** Verdicts for each of six RCTs, indicating number of assessors rating 'Yes' (Y), 'Unclear' (U), or 'No' (N) per domain

**Lewith, 2002 **[**26**]				
**Domain**	**Yes**	**Uncertain**	**No**	**Majority**

**I *(Rationale)***	3	1	4	N

**II *(Principles)***	7	1	0	Y

**III *(Practitioner)***	6	1	1	Y

**IV *(Outcome measure)***	6	2	0	Y

**V *(Sensitivity)***	8	0	0	Y

**VI *(Follow-up)***	5	1	2	Y

**Overall majority verdict**				**5Y+1N**

**Kainz, 1996 **[**27**]				

**Domain**	**Yes**	**Uncertain**	**No**	**Majority**

**I *(Rationale)***	6	2	0	Y

**II *(Principles)***	7	1	0	Y

**III *(Practitioner)***	5	3	0	Y

**IV *(Outcome measure)***	8	0	0	Y

**V *(Sensitivity)***	8	0	0	Y

**VI *(Follow-up)***	4	4	0	U*

**Overall majority verdict**				**5Y+1U**

**McCutcheon, 1996 **[28]				

**Domain**	**Yes**	**Uncertain**	**No**	**Majority**

**I *(Rationale)***	2	4	2	U

**II *(Principles)***	2	5	1	U

**III *(Practitioner)***	1	4	3	U

**IV *(Outcome measure)***	7	1	0	Y

**V *(Sensitivity)***	4	3	1	Y

**VI *(Follow-up)***	3	3	2	U*

**Overall majority verdict**				**2Y+4U**

**Bell, 2004 **[**29**]				

**Domain**	**Yes**	**Uncertain**	**No**	**Majority**

**I *(Rationale)***	8	0	0	Y

**II *(Principles)***	8	0	0	Y

**III *(Practitioner)***	8	0	0	Y

**IV *(Outcome measure)***	8	0	0	Y

**V *(Sensitivity)***	8	0	0	Y

**VI *(Follow-up)***	8	0	0	Y

**Overall majority verdict**				**6Y**

**Robertson, 2007 **[**30**]				

**Domain**	**Yes**	**Uncertain**	**No**	**Majority**

**I *(Rationale)***	6	1	1	Y

**II *(Principles)***	6	0	2	Y

**III *(Practitioner)***	4	2	2	Y

**IV *(Outcome measure)***	7	0	1	Y

**V *(Sensitivity)***	7	1	0	Y

**VI *(Follow-up)***	8	0	0	Y

**Overall majority verdict**				**6Y**

**Adler, 2009 **[**31**]				

**Domain**	**Yes**	**Uncertain**	**No**	**Majority**

**I *(Rationale)***	8	0	0	Y

**II *(Principles)***	8	0	0	Y

**III *(Practitioner)***	7	1	0	Y

**IV *(Outcome measure)***	8	0	0	Y

**V *(Sensitivity)***	8	0	0	Y

**VI *(Follow-up)***	6	1	1	Y

**Overall majority verdict**				**6Y**

*: In the case of tied majority between the two verdicts Y and U, the lower-ranked of the two (U) was used to reflect the rating of that domain: see domain VI (Kainz, McCutcheon)

## Discussion

Our international group of homeopathic practitioners/researchers has successfully identified a set of six key domains of RCTs that enables the appraisal of MVHT. Domains I-III address the 'homeopathicity' [12] of an RCT, while domains IV-VI address aspects of validity that are also germane to an RCT of any medical therapy. Even from the sample of just six homeopathy RCTs evaluated here by eight homeopathy specialists, it is evident that appraisal of key aspects of MVHT can be achieved using our proposed method.

According to accepted guidelines [34,35], the magnitude of the kappa statistic reflects inter-rater agreement as follows: κ = 0.01-0.20, slight, κ = 0.21-0.40, fair; κ = 0.41-0.60, moderate; κ = 0.61-0.80, substantial; κ = 0.81-0.99, almost perfect. Thus, concordance among assessors was 'fair' for domains I, III and VI (κ = 0.42-0.50), 'moderate' for domain II (κ = 0.68), and 'almost perfect' for domains IV and V (κ = 0.92-0.94). It remains to be seen whether disparity of opinion is intrinsic to the complexity of the underlying concepts and/or to any lack of clarity in the particular wording of the domain and criteria descriptions. It is usual, however, to find only limited concordance amongst independent assessors in the judgment of subjectively-based domains [35]. Nevertheless, if ambiguity of wording does become evident, a set of revised descriptions would be drafted and published.

In our use of the method, one trial (Lewith [26]) attained a majority of 'No', in domain I (rationale), which indicates that assessors regarded it unlikely that allergic asthma patients can be benefited by homeopathy *per se *and/or by the particular homeopathic regimen used in this trial; we would therefore class this trial overall as 'inadequate MVHT'. It is noteworthy that this trial did not fail our MVHT assessment on the basis of domain II and/or domain III (i.e. isopathy using house dust mite is consistent with homeopathic principles, and the study had suitably qualified and experienced homeopathic practitioner input). It was on the basis of perceived deficiencies in all three of the above attributes that led to previous adverse conclusions on this trial using non-formalised criteria for model validity [36]. Two trials (Kainz [27], McCutcheon [28]) were rated as 'unclear' in one or more domains and so may be classed overall as 'unclear MVHT'. The remaining three trials (Bell [29], Robertson [30], Adler [31]) each received a full complement of six 'Yes' majority verdicts and so may be classed overall as 'acceptable MVHT'.

This proposed method for appraising and summarising MVHT harmonises with the Cochrane Collaboration's technique for assessing risk-of-bias for internal validity. In the Cochrane approach, a trial is assessed against seven domains: sequence generation; allocation concealment; blinding of participants and personnel; blinding of outcome assessors; incomplete outcome data; freedom from selective reporting; freedom from other bias. For each domain, risk-of-bias is indicated by the response 'low risk', 'unclear risk' or 'high risk'; an overall classification of risk-of-bias per trial is then identified using the same terminology [3].

The formal appraisal of RCTs, as adopted by the Cochrane Collaboration for risk-of-bias (internal validity), is typically by *consensus *between or among assessors, not by majority. In our test of the new method, it was not our purpose to seek consensus but to identify a summary verdict per domain and thus to classify MVHT overall for each of six RCTs. In systematic review it is not assumed that each independent assessor will arrive initially at the same judgment per domain; the method for assessing MVHT would operate in the same manner, and we recommend using *three *assessors to account for diversity of homeopathic opinion. Despite the diverse nature of homeopathic practice, there is little reason to anticipate undue difficulty in the ability of three specialists - even those from differing 'schools' of homeopathic theory and practice - to achieve a reasonable consensus agreement in judging the relevant issues. As was intended in our approach here, the formal appraisal of MVHT would necessarily exclude the consideration of the trial results themselves, and it might be appropriate to conceal the Results and Discussion sections, as well as the authors' names, in the papers that are provided to assessors.

In initial consideration of our criteria for domain III (practitioner), some of our group expressed concern that published RCTs seldom include any account of the homeopathic prescribing rationale or approach to individualisation. After discussion, it was agreed that to insist on full information, such as repertorisation details, exceeds expectation of the current research literature in homeopathy as well as the typical word limits of journal papers. Moreover, we note that the *RedHot *guidelines include clear recommendations with respect to individualised homeopathic prescribing [19], and we encourage authors of RCTs to implement these high reporting standards. We adopted the more generic domain III judgmental criteria, as given in Table 1.

Additional discussion emerged about whether RCTs of homeopathy adequately take into account the complexity of individuals whose totality of signs and symptoms, rather than a named medical condition *per se*, is the basis of a homeopathic prescription. While we agreed this is an important facet of future research development, we accepted that the question whether clinical trials of homeopathy should place more emphasis on grouping together individuals with given characteristic signs and/or symptoms, rather than subjects with a specified medical condition, lies outside the terms of reference of the current project.

In judging domains IV-VI (outcome measure, sensitivity, follow-up) solely on a single 'main' outcome, we recognise that alternative outcome measures in the studies might have been selected and might have received different verdicts in our assessment of MVHT. Our approach here is consistent with the fact that a single, pre-determined, *primary *outcome is an important element in judging the validity of a trial. Our use of the WHO's International Classification of Functioning, Disability and Health, as became necessary for three of the six papers, has at least enabled us to make objective decisions on relative importance of health attributes within a clear hierarchical structure. For domain IV (whether the main outcome measure reflects the main effect expected of the intervention used), it is important that the consideration of 'expected main effect' also embraces the whole-person nature of the intervention and its associated outcome [37] - especially for homeopathy trials that involve individualised patient prescribing. Future assessments of MVHT of published trials should, where appropriate, include such 'whole-person' consideration, which might be achieved by extending the criteria by which 'main outcome measure' is selected [38]. This initiative might, in turn, assist the planning of future homeopathy trials by sharpening researchers' focus on the relevance of the outcome measures used.

Together with Cochrane-style assessment of risk-of-bias regarding internal validity, the assessment of external validity using the methods of either Bornhöft [4] or Jonas [11], and the *RedHot *guidelines for reporting [19], the domains and criteria we propose for appraising MVHT can enable a complete critical appraisal of appropriate RCTs in homeopathy. For reviews aiming to assess model validity in other types of RCT (e.g. prophylaxis rather than treatment) or in addressing a different research question (e.g. "Do molecular and ultra-molecular homeopathic potencies differ in their clinical effects?"), the domains/criteria we propose here are likely to require suitable adaptation. In any event, as suggested above, some modification of the currently proposed descriptions may be necessary in the light of experience in applying them in systematic reviews. With further suitable amendment, the approach we propose would also be relevant for assessing model validity in RCTs of complex interventions more generally, including other CAM disciplines.

## Conclusion

Six novel judgmental domains enabled MVHT to be assessed with 'fair' to 'almost perfect' concordance in each case. For the six RCTs examined, the method allowed MVHT to be classified overall as 'acceptable' in three, 'unclear' in two, and 'inadequate' in one. The MVHT assessment method should be applied in future systematic reviews of RCTs in homeopathy.

## Abbreviations

RCT: Randomised Controlled Trial; CAM: Complementary/Alternative Medicine; MVHT: Model Validity of Homeopathic Treatment; ISCHI: International Scientific Committee for Homeopathic Investigations.

## Competing interests

The authors declare that they have no competing interests.

## Authors' contributions

RTM conceived the study, devised the preliminary assessment domains, co-ordinated the email discussions leading to the refined set of domains, prepared the tabulations of assessments ready for statistical analysis, and drafted and finalised the manuscript. All other authors contributed to the round-the-table discussion and assessment of the preliminary domains, to the development of the refined set of domains, and to the editing of the manuscript for publication. Eight authors (PF, M-FB, JF, JJ, CN, MO, HR, MvW) took part in the appraisal of papers using the refined set of domains. All authors read and approved the final manuscript.

## Pre-publication history

The pre-publication history for this paper can be accessed here:

http://www.biomedcentral.com/1471-2288/12/49/prepub
